# DNA as grabbers and steerers of quantum emitters

**DOI:** 10.1515/nanoph-2022-0602

**Published:** 2022-11-14

**Authors:** YongDeok Cho, Sung Hun Park, Ji-Hyeok Huh, Ashwin Gopinath, Seungwoo Lee

**Affiliations:** KU-KIST Graduate School of Converging Science and Technology, Korea University, Seoul 02841, Republic of Korea; Department of Mechanical Engineering, Massachusetts Institute of Technology, Cambridge, MA 02139, USA; Department of Integrative Energy Engineering, Department of Biomicrosystem Technology, and KU Photonics Center, Korea University, Seoul 02841, Republic of Korea

**Keywords:** DNA, DNA origami placement, helicity, soft quantum emitters, Watson-Crick complementarity

## Abstract

The chemically synthesizable quantum emitters such as quantum dots (QDs), fluorescent nanodiamonds (FNDs), and organic fluorescent dyes can be integrated with an easy-to-craft quantum nanophotonic device, which would be readily developed by non-lithographic solution process. As a representative example, the solution dipping or casting of such soft quantum emitters on a flat metal layer and subsequent drop-casting of plasmonic nanoparticles can afford the quantum emitter-coupled plasmonic nanocavity (referred to as a nanoparticle-on-mirror (NPoM) cavity), allowing us for exploiting various quantum mechanical behaviors of light–matter interactions such as quantum electrodynamics (QED), strong coupling (e.g., Rabi splitting), and quantum mirage. This versatile, yet effective soft quantum nanophotonics would be further benefitted from a deterministic control over the positions and orientations of each individual quantum emitter, particularly at the molecule level of resolution. In this review, we will argue that DNA nanotechnology can provide a gold vista toward this end. A collective set of exotic characteristics of DNA molecules, including Watson-Crick complementarity and helical morphology, enables reliable grabbing of quantum emitters at the on-demand position and steering of their directors at the single molecular level. More critically, the recent advances in large-scale integration of DNA origami have pushed the reliance on the distinctly well-formed single device to the regime of the ultra-scale device arrays, which is critical for promoting the practically immediate applications of such soft quantum nanophotonics.

## 1 Introduction

The colloidal and molecular quantum emitters (MQEs) such as quantum dots (QDs) [1–5], organic fluorescent dyes [6–8], and fluorescent nanodiamonds (FNDs) [9–12] have become readily accessible by benefitting from the recent advances in their chemical synthetic routes. These go-to tools of quantum emitters have promoted the rapid democratization of quantum-mechanical studies on light–matter interactions for experts and newcomers alike [13–20]. For example, the weak and strong couplings between the quantum emitters and plasmonic cavities have been observed from the non-lithographically available plasmonic-molecular hybrid [21–24], in which the synthetic plasmonic nanoparticles (NPs) are simply put onto the fluorescent dyes on a flat metallic mirror. These are called NP-on-mirror (NPoM) cavities [21–30]. The dipping of a flat metallic layer in the fluorescent dye solutions and subsequent dropping of plasmonic NPs on it are all the required processes for the development of NPoM plasmonic cavity, which can be achieved even at room-temperature benchtop conditions without the dependence of any infrastructures (i.e., clean room, electron beam lithography facility, etching facility, and so on).

Such soft quantum nanophotonics can benefit from ever-increasingly improved accuracy in positioning and orientating emitters. For instance, the deterministic control over the relative positions and orientations of the electrically excited optical dipoles within the cavity is the key to the reliable achievements of a strong or weak coupling [21–24]. While considerable progress has been achieved this way, we will discuss in this review that DNA molecules can be unique enablers for grabbing and steering soft quantum emitters due to the following features.

First, the Watson and Crick complementarity of DNA enables the precise positioning of colloidal and MQEs, especially onto the DNA origami molecular pegboards, which are composed of massively parallelized DNA duplexes. It has been proved that about 5–8 nm resolution of such positioning is possible with the assistance of a flat sheet-typed 2D DNA origami [31–34]. This resolution can be further reduced to even Bohr radius by regulating the 3D geometrical space of two DNA origami slats [35]. Second, the helically defined major and minor grooves of DNA duplexes can serve as the orientation-controlled dockers of MQEs [36–42]. As with the first merit, DNA origami can serve a molecular pegboard, providing multiple molecular steerers of quantum emitters. Lastly, such DNA grabbers and steerers can be integrated over the large substrate (even wafer-scale) via the recently emergent DNA origami placement (DOP) technology [43–50]. The advent of DOP implies that on-demanding numbers of quantum emitters, whose orientations can be individually controlled, can be periodically or non-periodically assembled on a wide-scale wafer chip. In the following sections, we will summarize the previously reported seminal works toward this end and then, critically test whether the exotic abilities of DNA as molecular grabbers and steers of emitters can be advantageous over other competing options.

## 2 Watson–Crick complementarity and DNA duplexes

**Figure 1:**
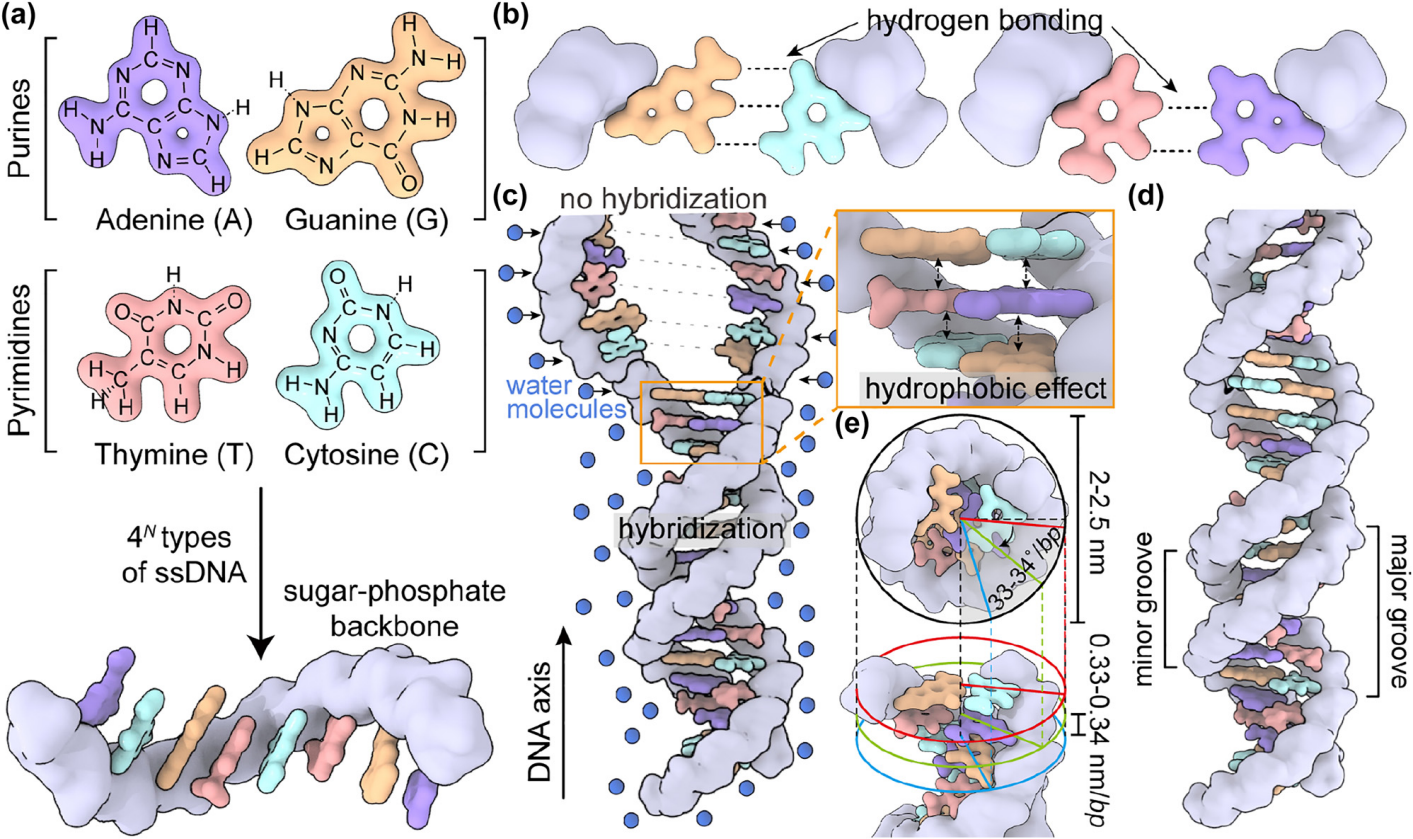
Schematic representations for fundamental of DNA, (a) the basic building block of DNA strand, composed of 4 different nucleotides including Adenine (A), Guanine (G), Thymine (T), and Cytosine (C) with sugar-phosphate backbone. (b) The different number of hydrogen binding sites between G with C and T with A respectively. (c) The hybridized *bp* can be stacked along the DNA axis. The unsolvable *bp* gets closer by surrounded water molecules through hydrophobic effect (in orange box) (d) entire scheme on twisted B-form DNA duplex. Along the helically twisted form, the major and minor groove have been labeled. (e) Top and perspective view of DNA duplex to show the width of 2–2.5 nm, rotational angle of 33∼34^°^/*bp* and distance of 0.33–0.34 nm/*bp*, respectively.

Single-stranded DNA (ssDNA) is a linear polymer, where 4 digits (A, T, G, C) of bases are sequentially grafted from a phosphate backbone with the intermediating of sugar molecules (Figure 1a). Thus, the possible numbers of a different ssDNA can be differentiated by a factor of 4^*N*^, where *N* indicates the base numbers in ssDNA (or monomer numbers of ssDNA). For example, *N* of 10 leads to over one million different ssDNA.

During the hybridization of two ssDNA molecules, all the possible hydrogen bonding sites of the bases of ssDNA are likely filled with their complementary counterparts. This is a thermodynamically favorable pathway for overcoming an entropic penalty caused by the hybridization of two complementary ssDNA molecules and for becoming an equilibrated state with a smaller enthalpy and the resultant Gibbs free energy compared with those of an unhybridized state [51, 52]. Toward this end, A and G specifically bind to T and C, respectively, because each pair retain the same hydrogen bonding sites (two for A–T; three for G–C) (Figure 1b) [53]. Otherwise, the empty sites for the hydrogen bonding inevitably remain such that the possible reduction of enthalpy is limited. Therefore, each specific sequence of ssDNA can be hybridized only with a complementary counterpart (Watson–Crick complementary binding).

The hybridized base pairs (*bp*) between two ssDNA can be axially stacked by a hydrophobic effect: water molecules likely push the unsolvable *bp* into a vertically stacked conformation (Figure 1c) [54]. During this process, the mechanical stresses, resulting from the electrostatic repulsions between the stacked *bp*, can be released by the rotational twisting of *bp* in a 1D helical fashion (Figure 1d). As a result, a helically featured duplex of DNA can be finally formed (B-form DNA) [53, 54]. The rotational angles and distances between *bp* are 33–34° and 0.33–0.34 nm, respectively, while the width of duplexes is about 2.0–2.5 nm (Figure 1e). Also, the major and minor grooves can be alternatively formed along the surfaces of duplexes (Figure 1d). As summarized in the following sections, such a collective set of molecular features of DNA (i.e., complementarity, helically rotated *bp*, and major/minor grooves) can afford the exotic abilities to grab and steer soft quantum emitters in a highly deterministic manner.

## 3 DNA origami as molecular pegboards

**Figure 2:**
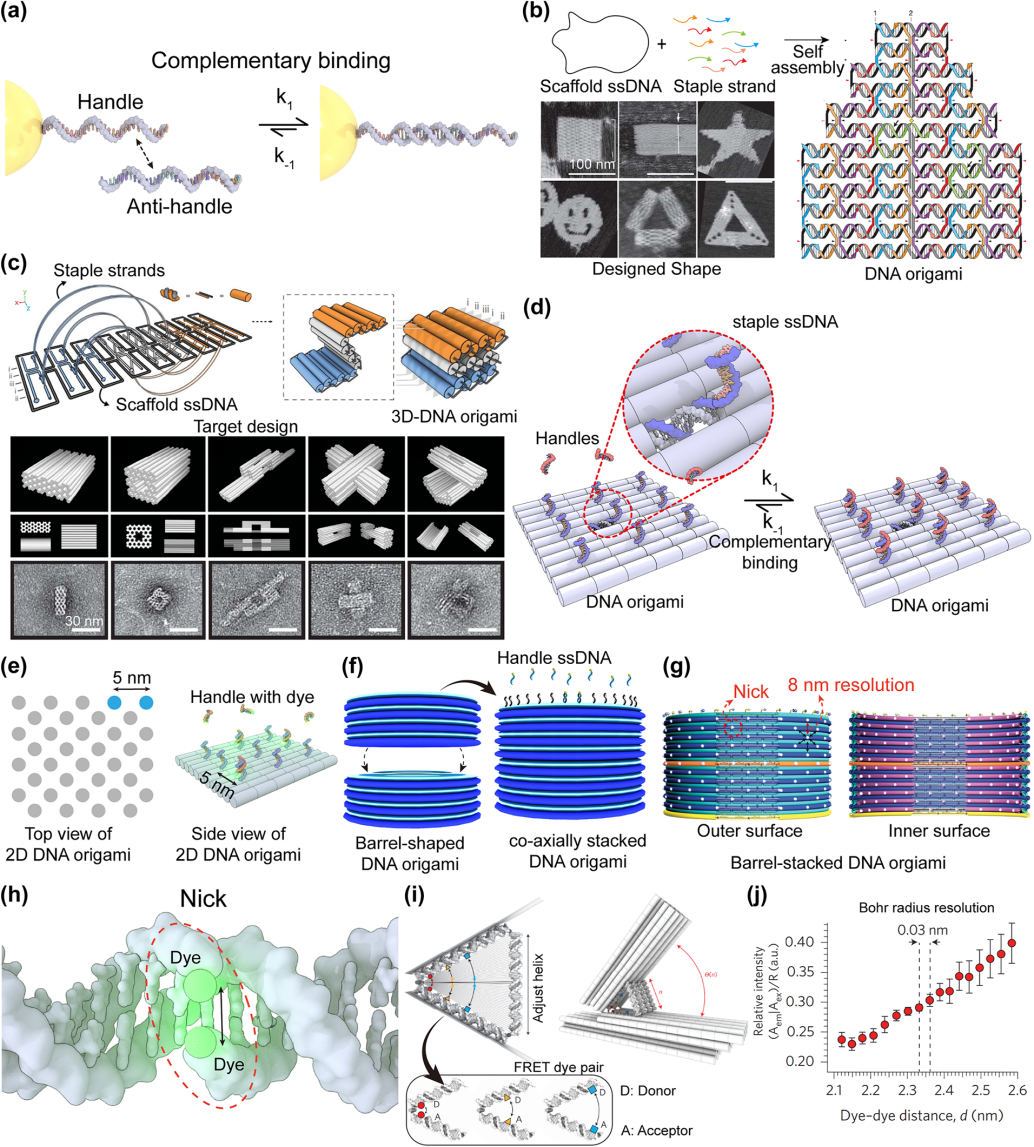
DNA origami for MQE’s pegboard, (a) the complementary binding of ssDNA decorated on the surface of AuNP with anti-handle. (b) Self-assembly of DNA origami with a scaffold ssDNA with various staple strands. The AFM images of various shapes including square, rectangle, star, disk with three holes, triangle with rectangular domain, and sharp triangle with trapezoidal domain [63]. (c) Schematic design of construction 3D DNA origami [64]. (d) The complementary binding mechanism between handles and anti-handles, hanging from the scaffold of the DNA origami (the gray strand). (e) The schematic representation for the positions of ssDNA anti-handles on DNA origami. The approximate distance between the adjacent ssDNA anti-handles is around ∼5 nm, which implies that the dye within handles can blink on DNA origami with ∼5 nm resolution. (f) Schematic design of two barrels coaxially stacked 3D DNA origami. With help of ssDNA, the fluorophore dyes can be positioned on to the specific sites. (g) Inner and outer surface of 3D barrel DNA origami. The fluorescent dye can be tagged on a nick with 8 nm resolution [77]. (h) Two dyes located on adjacent ‘nick’ within 1 nm. (i) DNA origami based high-resolution device for positioning two dyes by adjusting the distance of helix to induce the Förster resonance energy transfer (FRET) [35]. (j) The graph indicates the controllability of dye-to-dye distance within 0.03 nm resolution, which is comparable to Bohr radius. Part (b) adapted with permission from Ref. [63]. Part (c) adapted with permission from Ref. [64]. Part (g) adapted with permission from Ref. [77]. Parts (i) and (j) adapted with permission from Ref. [35].

As shown in Figure 2a, the various quantum emitters, decorated with ssDNA (i.e., handle DNA), can be grabbed by their complementary ssDNA (i.e., anti-handle DNA). Thus, the on-demand integration of anti-handle DNA onto a specific substrate is a priority for positioning quantum emitters with a nanoscale resolution. Until the middle of the 2000s, the lithographic integrations of ssDNA on a solid-state substrate had been the main research streams [55–62]. For instance, dip-pen nanolithography (DPN) enables the docking of the thiolated ssDNA onto an Au substrate with a nanopen-drawn, arbitrary geometry [59–62]. In general, any lithographic approach, however, would be impossible to achieve single molecular level controllability in terms of positioning ssDNA with a nanoscale resolution.

The advent of DNA origami in 2006 by Rothemund has effectively addressed such technical challenges [63]. A bunch of DNA duplexes can be parallelly interweaved via Watson–Crick complementary binding and then assembled into any desired 2D shapes of ∼100 nm scales (Figure 2b): folding ∼8000 base-ssDNA scaffold into the bundles of duplexes with on-demand shape and size, which can be molecularly programmed by the relatively short ssDNA staples (200–300 different numbers with 20–50 bases). The bundles of DNA duplex can be also programmed to be assembled into the 3D shapes without ruining the shape controllability (Figure 2c) [64–66].

Then, we can precisely decorate the surface of DNA origami with anti-handle ssDNA [67–73]. For example, half of a specific staple ssDNA, among others, can be programmed to hang out from the body of DNA origami, whereas the rest of it can be hybridized with scaffold DNA within the body of DNA origami (Figure 2d): generally, 4–9 base-based ssDNA has been used as an anti-handle. Therefore, the numbers of anti-handle ssDNA to be integrated on a DNA origami surface can be deterministically controlled at a single molecular level by programming the base sequence of staple ssDNA.

Indeed, ∼5 nm resolution of positioning each individual anti-handle ssDNA on a 2D single sheet of DNA origami has been proved, for example, by resolving fluorescence blinking of the handle ssDNA-grabbed dyes (Figure 2e) (i.e., super-resolution optical microscope imaging, empowered by DNA-based points accumulation for imaging in nanoscale tomography (DNA-PAINT) [31–34]. This 2D positioning of each individual molecule can be extended to a 3D space [74–77]. For example, the barrel-shaped DNA origami was co-axially stacked (Figure 2f) [77]; then, a few hundred fluorescent dyes can be independently rendered at their on-demand positions on the inner and outer surfaces of these DNA origami barrel-stacks (Figure 2g). In this case, ∼8 nm spatial resolution was experimentally addressed with DNA-PAINT [77].

One could beg the question: why were such experimentally addressed resolutions unable to be comparable to the characteristic scales of DNA duplexes (0.33–0.34 nm of the *bp* distance and 2.0–2.5 nm of duplex width)? As shown in Figure 2f, two anti-handle ssDNA can hang out each from two adjacent ends of staple ssDNA (or two adjacent *bp*). However, the hanging anti-handle ssDNA should undergo molecular pivoting and wobbling [51]; this molecular stochasticity in conjunction with a steric hindrance limits the sub-5 nm-level spatial resolution of MQEs. Note that for bigger quantum emitters such as FNDs and QDs, the achievable spatial resolution can be further sequestered particularly due to the steric hindrances.

One could imagine that the fluorescent dyes can be inserted directly within the specific duplex of DNA origami [31], [32], [33], [34, 39], [40], [41, 77]. In particular, the recent advance in a polymerase chain reaction (PCR) enables the covalent linking of various fluorescent dyes to one end of the ssDNA oligo. As such, the fluorescent dyes can be located selectively at the end of the designated staple DNA (generally referred to as “nick”), while other nicks remain intact (Figure 2g) [77]. Or two different dyes can be located at each nearest neighboring end of a specific staple DNA (Figure 2h) [33]. This non-use of anti-handle DNA can push two dyes to be faced with a 1 nm or sub-nm distance. However, in this case, the control over the on-demand location of the fluorescent dyes is unable to achieve, as a lot of nicks within DNA origami tend to be randomly distributed.

In 2016, Funke and Dietz partly overcame such a technical challenge by the rational design of a DNA origami ruler [35]. As presented in Figure 2i, the edges of two DNA origami plates are connected by a hinge (i.e., ssDNA backbone), while the tuning of the adjuster helix length defines the rotational angles between them. Two fluorescent dyes are programmed to be located each at the inner facets of the bottom and inclined DNA origami plates. Therefore, the control over the distance between two adjacent dyes can follow the theorem of intersecting lines, which in turn decouples the positioning degree of freedom from the geometrical constraints of DNA duplexes, mentioned above. Also, all the components of such DNA origami rulers are based on a relatively rigid bundle of DNA duplexes rather than ssDNA, making them robust against thermal fluctuation-driven molecular stochasticity. According to the base numbers of the adjuster helix, the distance between two fluorescent dyes was controlled from 1.5 nm to 9 nm, especially with a displacement step of ∼0.03 nm (Figure 2j), which is comparable to the Bohr radius.

**Figure 3:**
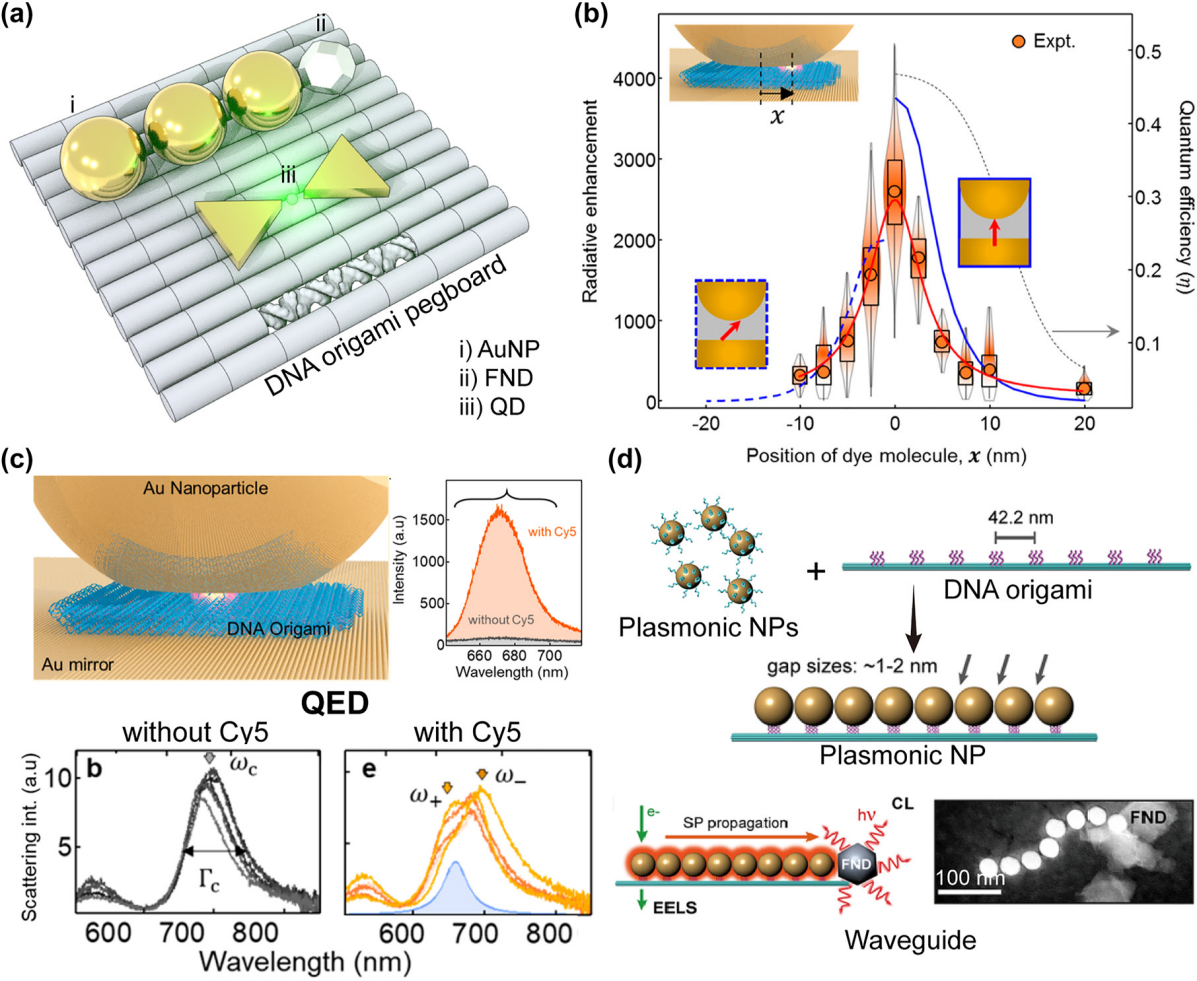
The usage of DNA origami in nanophotonics, (a) DNA origami as a molecular pegboard. Various MQEs including AuNP, FND, and QDs are placed on DNA origami through complementary binding. (b) Molecular quantum efficiency variation with respect to molecular positioning within NPoM cavity system. The dye location varies via DNA origami positioning. (c) Plasmonic cavity system via NPoM with single molecule within DNA origami [23]. The dye within hot spot strongly interact with cavity system results strong coupling (i.e., Rabi splitting). (d) Plasmonic waveguide mediated by DNA origami helix bundles [85]. Through surface plasmon (SP) propagation, the energy is transferred to FND with emission signal on the other side. Bottom panel shows experimentally realization of plasmonic waveguide. Parts (b) and (c) adapted with permission from Ref. [23]. Part (d) adapted with permission from Ref. [85].

In the last part of this section, we will remark on the capability of DNA origami to heterogeneously integrate such soft quantum emitters with nanophotonic elements. As a representative example, plasmonic NPs have been co-assembled with fluorescent dyes, QDs, and FNDs [78] (Figure 3a). DNA origami pegboards can push these quantum emitters to be deterministically positioned into the hot spots of plasmonic cavities (plasmonic nanogap) [22], [23], [24, 79], [80], [81], [82], [83], which were assembled by the molecularly programmed dimerization of two Au NPs (nanospheres or nanoprisms) or formation of NPoM. Thus, the emission efficiency (*η*) from such plasmonic cavity-coupled quantum emitters has been precisely tuned (Figure 3b) [79–83] or quantum electrodynamics (QED) was experimentally observed (Figure 3c) [22–24]. Also, the linear chain of plasmonic NPs, assembled on the DNA origami (the long tube motif based on a six-helix bundle), acts as a waveguide [84, 85]. As such, FND, assembled right after the plasmonic NP chain on DNA origami, can emit the photon to be guided at the nanoscale geometry (Figure 3d) [85].

## 4 Orientational control of MQEs

The spatial alignment of the optical dipole of the quantum emitter with the surrounding optical cavity has served as the determinant for boosting light–matter interaction. Indeed, several efforts on absolute control of the long axis of molecular emitters over hot spots within the optical cavity have long been pursued. The use of host-guest chemistry of cucurbit molecules was a pioneering approach for vertically aligning molecular quantum emitters (methylene blue (MB)), whose optical dipole can be near perfectly parallel to the electrically squeezed dipolar field in the plasmonic NPoM [22]. However, the host-guest alignment approach still faced a lack of the full degree of freedom on 3D orientation control.

**Figure 4:**
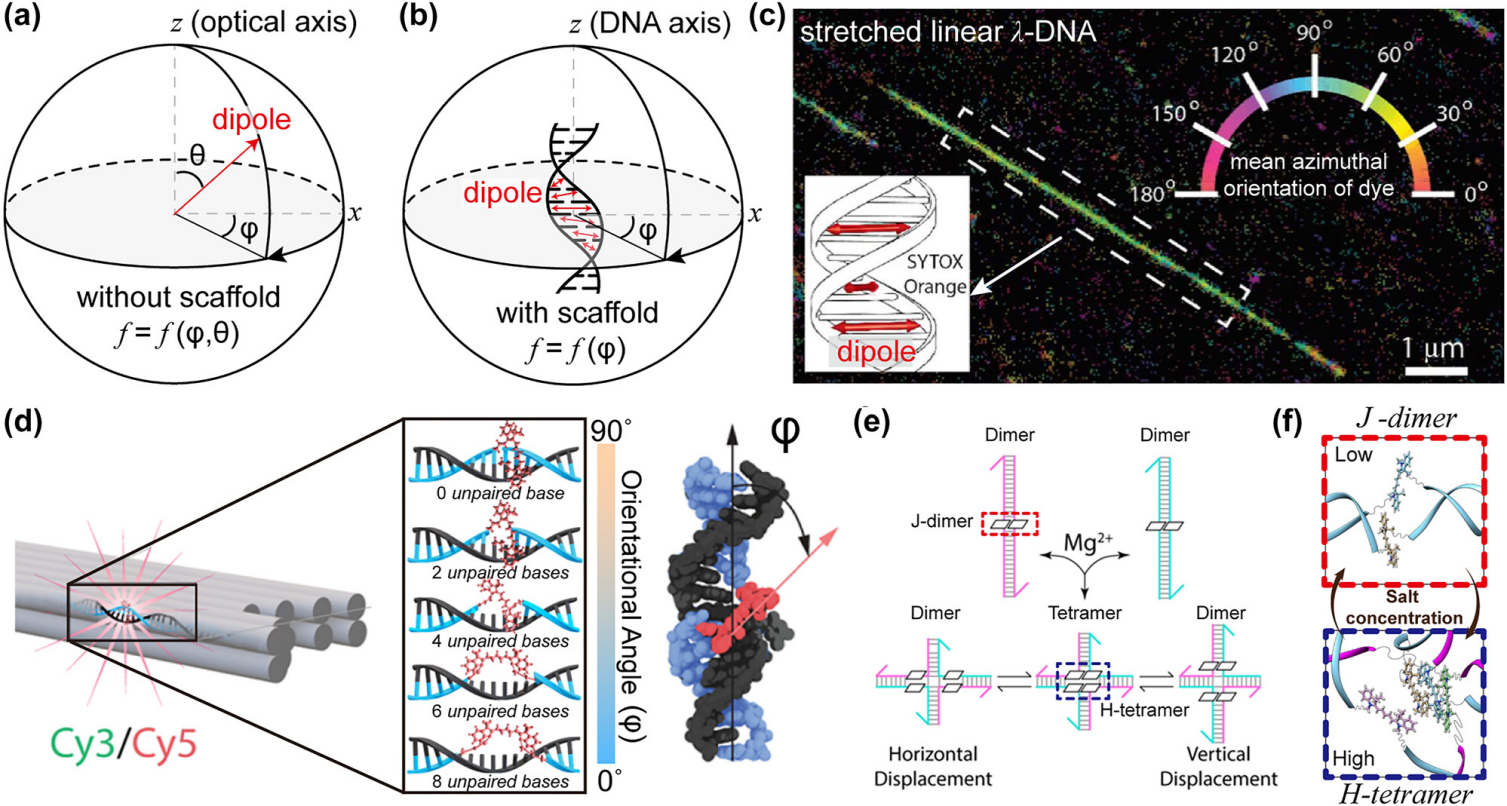
The rotational degree of freedom for dye molecules and restriction of dye orientation within DNA duplex. (a) The representation of polar system for free the free molecules with azimuthal angle (*φ*) and polar tilt (*θ*). (b) The representation of polar system for constrained dyes in dsDNA. (c) Super-resolved image for stretched linear λ-DNA, intercalated with SYTOX orange (inset) [36]. Color bar denotes the mean azimuthal orientation of dye molecules. Measured resolution is within 30 nm voxel. (d) Different dye orientation according to number of 0–8 unpaired bases [41]. Cy3/Cy5 is doubly linked to the nick of DNA strands. (e) The salt-driven configuration changes between two J-dimers and H-tetramer via a four-way branch migration [42]. (f) Illustrative representation of J-dimer (heat-to-tail arrangement) and H-tetramer (parallel arrangement) [42]. Both configurations are reversibly converted depending on the salt concentration. Part (c) adapted with permission from Ref. [36]. Part (d) adapted with permission from Ref. [41]. Parts (e) and (f) adapted with permission from Ref. [42].

Hence, new technical approaches have been developed with the help of the usage of polarized optical microscopy with DNA duplex strands [36–38]. In 2016, Moerner et al. reported the tour de force method to vividly visualize the measurement of single molecule orientation [36]. One can imagine that a single quantum emitter can freely rotate and wobble without any restriction, which gives difficulty in analyzing the emission of a single quantum emitter. Indeed, the uncertainty of molecular position enforces one to define unknown two parameters: its azimuthal orientation phi (*φ*), and its polar tilt theta (*θ*) with respect to the optical axis (*z*) (see Figure 4a) to fully characterize the emission of a single molecule. However, this method requires measurement of polarized single-molecule intensity on at least three more polarization axes [86–88]. To simplify the matter, the single molecule has been spatially constrained within a DNA duplex as seen in Figure 4b. This constraint consequently aligns a single molecule to be perpendicularly positioned along the DNA duplex axis. The single fluorescence molecule, SYTOX Orange dye, inserted within adjacent *bp*, has been measured with super-resolved microscopy as shown in Figure 4c. By rotating the excitation of polarization, the emission intensity of dye molecules will be changed proportionally to the angle between absorption dipole moment $\vec{\mu }$ of emitter with polarization vector of $\vec{E}$ of excited light [89]. The experimental implementation was then visualized with **λ**-DNA strands with a localization precision of 27 nm (Figure 4c). The color mapping of the upper right-side shows the azimuthal angle distributions of dyes with respect to the DNA duplex alignment.

More precise angle control of dye molecules comes with DNA origami techniques [40, 41]. First, the spatial positions of MQEs (e.g., Cy5 and Cy3) can be defined by a covalent linkage of them at the end of the designated staple DNA [41]. Then, as presented at the top of Figure 4d, two ends of MQEs can be covalently linked to the nearest neighboring each end of two staple DNA (i.e., nick position). In this case, the long axis of MQE likely becomes perpendicular to the duplexes in DNA origami (*φ* of 90°). This orientation can switch off to *φ* of 0° by bidirectional stretching of MQEs (from top to bottom of Figure 4d). The gradual shortening of the numbers of ssDNA bases from the nick position can stretch MQE along the direction of the duplex, while the spatial position of MQEs in DNA origami remains intact.

Apart from individual molecular orientational design, a dimer formed by two-dye aggregation has gained much attention due to its potential usage with intriguing optical phenomena. One can imagine J- and H-aggregation with dye–dye interaction and their optical features [90–92], where it can be a key mechanism in terms of understanding coherent exciton delocalization [93–96]. Recent work has shown a DNA templated mediated dye aggregation system [42], where Cy5 dye is internally functionalized within DNA oligomers. Figure 4e shows the Cy5 dyes arranged in a head-to-tail form, J-aggregate, within a DNA duplex under low concentrations. However, once the concentration of salt ions increases, the J-dimer goes a significant internal rearrangement to an H-type tetramer with two DNA duplexes with four armed junctions (i.e., Holiday junction). Note that those geometrical configurations have been depicted in Figure 4f. This demonstration has proven that changing the salt concentration can control two aggregated states throughout dye–dye interactions.

## 5 To be, or not to be periodic over the large area

DNA origami is the particulate pegboards, initially floating in a fluid. Each DNA origami has been simply docked onto the solid-state substrate (e.g., mica surface) via electrostatic interactions, and then structurally and optically characterized (e.g., DNA-PAINT, atomic force microscope (AFM) imaging, and so on) [31–34]. In this routinely used protocol, their orientational and positional orderings are out-of-control, severally limiting the usage of such DNA origami (and related juxtaposed quantum emitters) to the discretely well-formed device.

**Figure 5:**
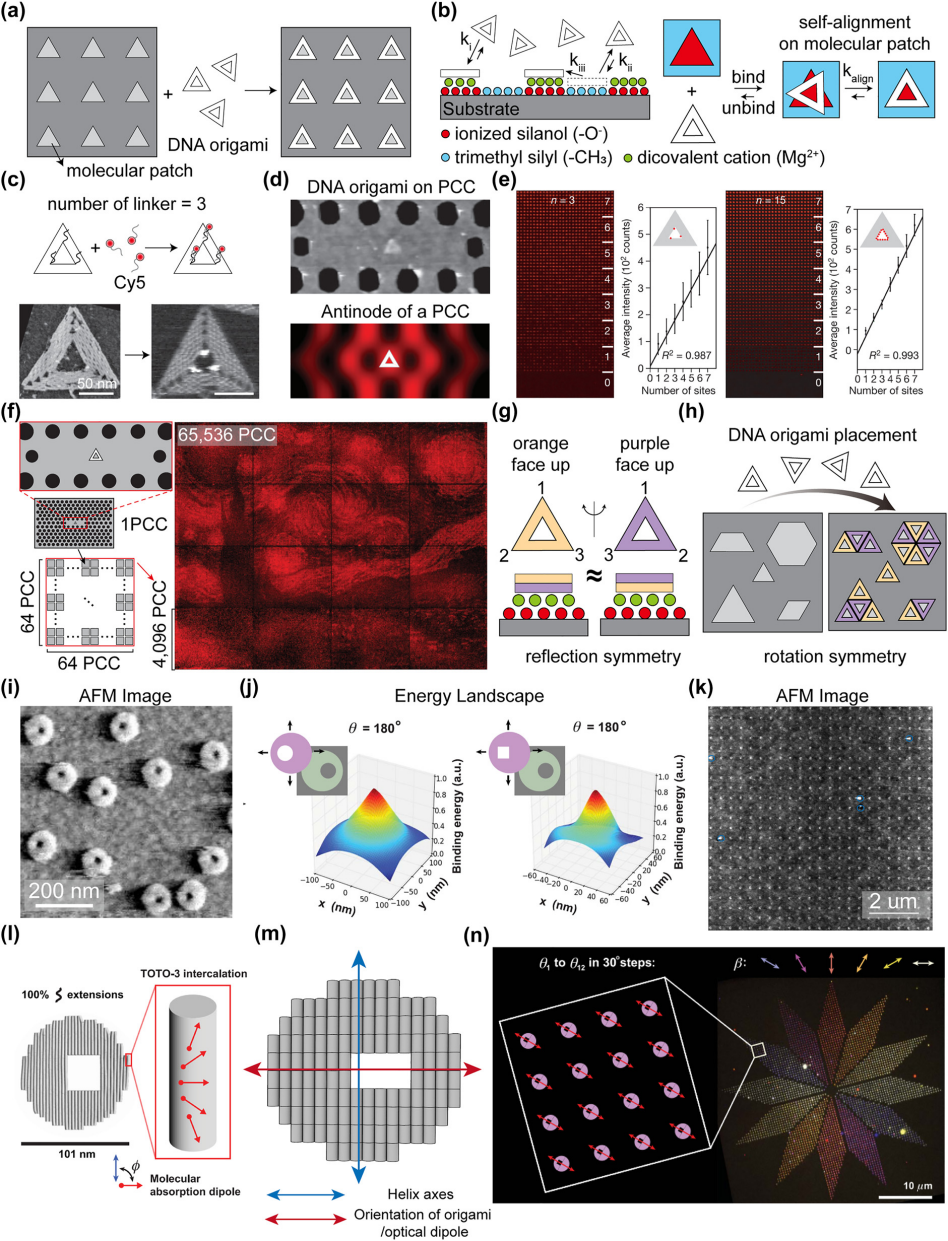
Controlling the molecular position and orientation on wafer-scale area. (a) The molecular patches, which are compatible with the DNA origami sizes and shapes, can roughly guide molecular position and orientation. (b) Left: Magnesium ion mediated DNA origami placement on the binding sites through direct docking (k_i_), indirect docking (k_ii_) and surface diffusion (k_iii_) from electron-poor site to electron-rich site. Right: The DNA origami lying on a negatively charged patch undergoes self-alignment (k_align_) for minimizing the free energy. (c) Conjugation of dye molecules on the triangular DNA origami [48]. (d) Cy5-embedded DNA origami placed on antinode of PCC [48]. (e) Comparison for fluorescence intensity with different number of DNA origami in antinodes and precisely tuning the quantum yield with *n* = 3 and *n* = 15 Cy5/origami [48]. (f) Van Gogh’s *The Starry Night* with 65,536 cavities [48]. (g–h) Reflection symmetry (g) and rotation symmetry (h) in the equilateral triangular DNA origami. (i) AFM image of *small moons* [50]. (j) Section of binding energy landscape for perfectly placed the ideal shape and experimental shape of *small moon* on molecular patch (at *θ* = 180°) [50]. Color is encoded from high (red) and low (blue) binding energies. (k) AFM image of *small moons* placed on a square array. Blue markers indicate no analyzed sites. (l) Schematic representation for rotations of the intercalated TOTO-3s′ absorption dipole in DNA helixes. (m) The representation of helix axes and orientation of origami/optical dipole. (n) Polarimeter image of 3456 *small moon* splits into 12 rhomboidal arrays [50]. For each rhomboidal array section, the optical dipole is rotated with 30° step, $\vec{\mu }$ of *small moons* in each section are headed outside (see inset). The $\vec{E}$ at maximized intensity is represented as *β*. Parts (c)–(f) adapted with permission from Ref. [48]. Parts (i)–(l) and (n) adapted with permission from Ref. [50].

To technically translate DNA origami-based quantum nanophotonic devices into practically viable regimes (e.g., quantum plasmonic sensing chip), a wide-scale arraying of each particulate DNA origami (at least the chip-scale integration) with a well-controlled translational position and orientation should be a pivotal step; Gopinath and Rothemund effectively tackled this technological challenge by establishing an ideal design of DNA origami and the associated experimental protocols for DOP [46]. The regularly arrayed molecular patches, whose shape and size can be commensurate with the DNA origami motif, can be docking sites of the floating DNA origami with deterministically defined positions and orientations (Figure 5a).

Generally, DNA origami can be directly adsorbed onto the positively charged patches via electrostatic attraction since their surface is negatively charged (i.e., the intrinsic nature of phosphate backbones). Or the (pseudo) or covalent bonding between the thiolated strand and gold substrate can dock the DNA origami onto the substrate. However, these strong electrostatic attractions and thiol-Au bondings lead to the diffusion-limited, non-equilibrium fixing of DNA origami, consequently resulting in a poor yield in terms of positional and orientational accuracy [97–99]. A moderate interaction between DNA origami and substrate can give an additional opportunity for repositioning and realigning, such that it can bring DNA origami to a near-equilibrium position with a sufficiently high yield. Toward this, it turned out that the mediation by the positive ions (e.g., magnesium ions) between the negatively charged molecular patch (e.g., ionized silanol) and DNA origami can facilitate a series of binding, unbinding, and re-binding until the DNA origami becomes perfectly self-aligned with a commensurate molecular patch (Figure 5b). Herein, the molecular patches can be developed by electron beam lithography or nanoimprint lithography.

In the initial developmental stages, DOP was based on the equilateral triangular frame of the DNA origami [43–48]. For example, as shown in Figure 5c, the triangular DNA origami, which is decorated with a controlled number of fluorescent dyes via the handle and anti-handle complementary binding, can be deterministically placed at the on-demand position of the photonic crystal cavity (PCC) [48]: it is visible that the triangular DNA origami can be deterministically placed on the antinodes of PCC (see Figure 5d). With respect to the numbers of DNA origami to be on the antinodes of PCC, the quantum yield of fluorescent dyes (herein, Cy5) can be precisely tuned (Figure 5e). More importantly, it was proven that over a million numbers of DNA origami-fluorescent dye hybrids were simultaneously placed each on the molecularly defined, ∼65,000 different positions of PCC (Figure 5f), directly evidencing the chip-scale applicability of DOP.

However, this triangular DNA origami motif should have an equal probability for both upper and lower side placements (Figure 5g). Additionally, the triangular frame retains a rotational symmetry, implying that a 3-fold or 6-fold symmetric alignment between DNA origami and underneath molecular patches is possible (Figure 5h). Therefore, a broken-rotational symmetry is critical to achieving an absolute and arbitrary orientation of DNA origami (also, associated quantum emitters).

Gopinath and Rothemund found that a circular disk shape with an offset small hole (so-called *small moon*) among the vast variety of broken-rotational symmetries can be an optimal design of DOP (Figure 5i) [50]. In particular, the rounded outer of the disk rather than the sharp vertices or edges of the triangular motif can promote the refixing of alignment throughout the binding, unbinding, and re-binding of DNA origami (Figure 5j), such that the yield of DOP with an absolute and arbitrary orientation can be sufficiently enough over the large-area, as presented in AFM imaging (Figure 5k). This scalability in DOP was further highlighted by the wide view of fluorescent emission imaging as follows.

Once MQEs (e.g., TOTO-3 fluorescent dyes) are intercalated along the duplexes of DNA, their orientation along the DNA helix is approximately estimated at around 61° to 90° (Figure 5l). However, the summation of dipole from multiple dyes could result in a strong anisotropic net dipole [100]. Then, a *small moon* motif of DNA origami can be designed to consist of a uniaxially extended 2D bundle of duplexes, as shown in Figure 5m. Therefore, unidirectional dipolar emission is uniformly possible from each *small moon*-fluorescent dye hybrid by the illumination with a linear polarized light: the emission intensity is maximized, when the incoming linear polarization is parallel to the optical dipole. As presented in Figure 5n, total 12 rhomboidal pixels were radially integrated with a step rotation angle of 30°; each rhomboid was uniformly filled with the differently oriented subpixels of the *small moon* (∼288 numbers of *small moon* were regularly placed in each rhomboid). Under the illumination of a linearly polarized excitation, each rhomboid appeared a uniform intensity of fluorescence, which was differentiated according to the angle between linear polarization and optical dipoles.

## 6 Concluding remarks: perspectives and limitations

Taken together, we have shown that DNA nanotechnology, especially based on DNA origami, can afford a deterministic platform for the integration of soft quantum emitters. Across the QD, FNDs, and fluorescent dyes, the positional ordering of these quantum emitters with the resolution of a few nanometers is possible with the assistance of DNA origami pegboards; DOP leverages this localized ordering on the DNA origami to the wide-scale regular integrations, promoting a chip-scale application of quantum nanophotonics. Limited on a molecular quantum emitter (fluorescent dye), we can arbitrarily encode an absolute orientation of optical dipoles in each different DNA origami, which can be readily arrayed also by DOP. It is noteworthy that this wide-scale control over the absolute and arbitrary orientations of MQEs would be a challenge to achieve with other nanofabrication. Given the beauty of DNA as grabbers and steers of quantum emitters, we could envision the future research direction as follows.

Regarding the application of quantum nanophotonic devices, the realistic on-chip transporting and manipulating of single photon emission is yet to be proven. Toward this end, the DOP with an absolute orientation of emitters needs to be performed directly on a practical quantum photonic device such as a waveguide and detector-integrated system instead of a simple flat wafer. The robustness of DOP light source against the long-term operation of a quantum nanophotonic device could be also problematic as DNA is soft and even denaturable over 40 °C [101, 102], low salt conditions [103], and non-aqueous environments [104], and disturbance or quenching effect on the emitter due to surface modification [105]. Possibly, the conformal silicification on DNA origami could be the solution to the softness-driven weakness of DNA origami quantum emitters [106–109].

Also, the massive and deterministic placement of plasmonic NPs on the arrays of quantum-emitter-embedded DNA origami is out of reach thus far. In general, the dynamics of soft matters get slower as their characteristic length becomes larger. Therefore, the self-assembly of plasmonic NPs is much more error-prone compared with that of the pure DNA molecules; implying that a final yield of DNA origami-based NPoM arrays would drastically fall down. Recently, the thermodynamic understanding of DNA oligo-coated colloidal NPs and their self-assembly undertook considerable progress [110–115], bringing DNA origami-plasmonic NP assemblies closer to the equilibrium assembly [109, 116], [117], [118], [119], [120], [121]. However, there is still a long way to go for chip-scale integration.

Without their heterogeneous integration with quantum nanophotonic structures, DNA origami-emitter hybrids themselves could be useful for the quantum light source. The model of superradiance, conceived by Dicke in 1954 [122], could be a representative example, which could be accessible solely with DNA origami-emitter hybrids. The appropriate arrays of optical dipoles can collectively interact with each other, such that coherently radiative emission from the arrayed emitters is possible [123–125]. In this superradiance condition, the radiative intensity and decay rate can be boosted by a factor of *M*^2^ rather than *M*, where *M* indicates the number of emitters. Overall, the appropriate alignment and spatial control of MQEs on the DNA origami pegboards could address the goal of purely molecular superradiance; however, it remains challenging.
